# Identification and validation of respiratory virus immunization using natural language processing

**DOI:** 10.3389/fdgth.2026.1733630

**Published:** 2026-02-02

**Authors:** Kevin A. Wilson, John J. Riddles, Andrew C. Hill, Elizabeth A. Bassett, Mengshi Zhou, Michelle Barron, Catia Chavez, Rahul Shrivastava, Anil Battalahalli, Daniel Chacreton, Ethan Moran, Elizabeth Rowley, Zachary A. Weber, Lawrence Reichle, Sarah W. Ball, Amanda B. Payne, Jennifer DeCuir, Ruth Link-Gelles, Toan C. Ong

**Affiliations:** 1 Westat, Bethesda, MD, United States; 2 Colorado School of Public Health and ACCORDS School of Medicine, University of Colorado Anschutz Medical Campus, Aurora, CO, United States; 3 Coronavirus and Other Respiratory Viruses Division, National Center for Immunization and Respiratory Diseases, Centers for Disease Control and Prevention, Atlanta, GA, United States; 4 Influenza Division, National Center for Immunization and Respiratory Diseases, Centers for Disease Control and Prevention, Atlanta, GA, United States; 5 United States Public Health Service Commissioned Corps, Rockville, MD, United States

**Keywords:** COVID-19, electronic health records, immunization verification, influenza, natural language processing (NLP), respiratory syncytial virus (RSV), rule-based methods, vaccine verification

## Abstract

**Introduction:**

Electronic health record (EHR)-based research often relies on structured data elements, such as ICD-10-CM and CPT codes, to identify clinical diagnoses and procedures. However, some information, such as the administration of immunizations, may be captured more reliably in the text-based narrative sections of the patient's record. We developed a rule-based natural language processing (NLP) algorithm to identify the administration of immunizations for COVID-19, influenza, and RSV using a combination of synthetic and publicly available data.

**Methods:**

After applying standard NLP processing techniques to clean and standardize the text, we implemented a multi-stage, rule-based algorithm. We applied a dictionary of general keywords to identify potential immunizations, and a set of specific keywords, which leveraged grammatical dependencies in the text, to increase accuracy. We implemented additional rules to account for negation and immunization recommendations. The algorithm was applied to a sample of 20,000 patients from the study population. We measured performance by conducting a manual review of 400 individual notes and assessing concurrence with structured data, using precision and recall as evaluation metrics.

**Results:**

In the first evaluation, which compared the performance of the algorithm with an independent test dataset using manual clinical review, precision was 71% and recall was 97% for COVID-19 immunization; 91% and 92% for Influenza; and 57% and 96% for RSV. In a second evaluation using structured data as the gold standard (i.e., ICD-10-CM, CPT, and CVX codes), precision was 72% and recall was 9% for COVID-19 immunization; 71% and 12% for Influenza; and for RSV, precision was 78% and recall was 10%.

**Discussion:**

We demonstrated the effectiveness of NLP methods in identifying immunizations from EHR. High precision and recall for COVID-19 and influenza immunizations suggest that the algorithm can effectively identify immunization references when they are present in the text; however, low recall when compared to the structured data suggests that there are many more immunizations in the structured data not present in the text. Thus, the algorithm has specialized utility for augmenting immunization records using text data from individual notes; however, the algorithm's extensibility and generalizability can serve as a framework for future EHR-based research.

## 1 Introduction

Natural language processing (NLP) is a growing area of research that aims to develop algorithms and models capable of processing and decoding unstructured, human-generated text. In recent years, there has been interest in using NLP to analyze unstructured data within Electronic Health Record (EHR) systems (1). Unlike structured data, which consists of data organized into predefined data fields, typically categorical or numerical values, unstructured data consists of free text or fields without a predefined format. Although structured EHR sections contain most health information for individuals, often defined by standardized data elements such as ICD-10-CM diagnosis and procedure codes, a significant proportion of this information is contained within unstructured sections, such as physician notes (2). This poses a challenge to researchers who rely solely on structured EHR sections for data analysis. We developed NLP methods to extract critical data from text-based narrative sections of the patient's record, using immunization^1^ administration as a specific use case.

NLP methods take various forms and can employ a rule-based approach, machine learning (ML) models, or other artificial intelligence (AI) techniques, depending on the research needs of a specific project (2). In a rule-based model, experts develop a set of keywords and a series of rules that contextually detect terms in a sentence. In contrast, an ML model uses a preexisting training dataset to predict criteria or labels in a test dataset (3). Rule-based systems have been widely implemented for NLP tasks, such as computational phenotyping and clinical decision support; rule-based systems have been successfully developed for information extraction (3–9). Notably, Deady et al. (10) employed rule-based NLP methods to extract influenza vaccination data from clinical notes, resulting in a 16.8% increase in captured vaccinations.

Researchers have applied rules and model-based NLP methods across a range of therapeutic areas, including identification of bleeding events (11–13, 37), chronic cough (14), surgical site infections (15), prediction of falls (16), back pain (17), Hepatitis C (18), and a range of mental health issues (19, 20). The application of NLP methods has been valuable in situations where traditional diagnosis codes do not capture the disease of interest, such as the identification of diseases in undiagnosed populations and in situations where ICD 10-CM diagnosis and procedure codes are not specific enough to accurately identify the disease population (14, 18, 38). NLP methods have also been effective in identifying early predictors and longitudinal progression of disease and provide an alternative to labor-intensive chart review (16, 18). Rule-based methods are popular and effective because they do not require labeled training data (11, 14) and are able to algorithmically detect modifiers, such as negation, self-diagnosis, and affirmation (14, 16, 19). Methods range from relatively simple regular expressions to algorithms that incorporate semantic mappings, grammatical structure, and clinical terminologies (15). Rule-based methods have also been used in combination with ML approaches, including as a preprocessing step to generate labeled training data (17) and for identification of modifiers (12).

The success of previous studies using a rule-based NLP model to extract immunizations from text-based EHR sections guided our research efforts. Moreover, an analysis of NLP research on unstructured EHR data revealed that rule-based NLP models significantly outperform ML approaches while also providing increased simplicity, transparency, and interpretability (8). During an exploratory phase, preceding the extraction of EHR study data, we developed preliminary models based on publicly available EHR data using the Medical Information Mart for Intensive Care, version four dataset (MIMIC-IV) (9). Compared to a less malleable ML-based model, a rule-based approach provided increased flexibility in further developing our approach in this vaccine administration setting.

Respiratory viruses, such as SARS-CoV-2, influenza, and respiratory syncytial virus (RSV), remain persistent contributors of morbidity and mortality within the United States (21–23). As such, tracking data related to immunization coverage, effectiveness, and severe disease outcomes by immunization status are crucial in supporting public health efforts targeting these viruses (24, 25, 27). The need for a rapid public health response increases the reliance on timely and accurate EHR data. Vaccine effectiveness (VE) estimates support public health programs and policies by routinely collecting and synthesizing data on respiratory illnesses and associated outcomes in vaccinated and unvaccinated subjects, and publishing timely statistical analyses (26, 28–30). As pandemic-era regulations requiring providers to report immunization data to state and local immunization information systems have expired, there may be an impact on the reliability and completeness of immunization history data. Furthermore, structured EHR data may not reflect immunizations received in locations outside of healthcare networks, including unaffiliated clinical practices, pharmacies, and workplaces (10). Due to the importance of consistent and reliable immunization data for estimating VE and assessing immunization coverage, we investigated and developed methods to extract additional immunization data from relevant text-based EHR sections for study participants.

This study specifically examines the use and optimization of a rule-based NLP algorithm to identify and characterize immunization events in unstructured EHR sections, and broadly considers the application of a rule-based NLP algorithm to capture other measures and characteristics that are not reliant on ICD-10-CM diagnosis and procedure codes.

## 2 Materials and methods

### 2.1 Study population

The population for this study consisted of patients who received care within the University of Colorado Healthcare System and had available clinical note data within the study period, from August 1, 2023, to December 31, 2023. This patient population (*N* = 40,588) was initially identified as part of the VISION network, for which the methodology has been well established in the literature (28, 31, 32). The population was associated with 357,877 notes across three care settings. Specifically, 254,926 notes (71%) were recorded in the inpatient setting, 55,901 (16%) in the emergency department/urgent care setting, and 47,050 (13%) in the outpatient setting. This activity was reviewed by CDC and was conducted consistent with applicable federal law and CDC policy.^2^

### 2.2 Sample

We selected a stratified, systematic sample of 20,000 patients from the study population (*N* = 40,588). The sample was stratified by presence of structured immunization records, testing results, and immunocompromised status. Within each stratum, patients were sorted by age and sampled at even intervals. To ensure representativeness and reasonable sample sizes within different demographics, we stratified the sample by several variables of interest: immunization status in the structured EHR data for COVID-19, influenza, and RSV; pathogenic test result; age group; RSV immunization eligibility; and immunocompromised status. Table 1 presents the definitions for each variable. Notably, to be included in the sample, a patient had to have test results for at least one pathogen (i.e., SARS-CoV-2, influenza, RSV), although they could have missing test results for one or two pathogens. A preliminary review indicated that progress notes were likely to contain more relevant information than other note types, so notes were restricted to progress notes. Of 20,000 patients, we included 18,488 patients in the downstream analysis due to the availability of progress notes. From this sample, we selected three overlapping sub-samples to implement and evaluate separate rules for COVID-19, influenza, and RSV immunization. For the sub-sample, a random selection of patients was drawn from different strata defined by the presence of structured vaccination records, as well as presence/absence of vaccinations identified by an early prototype of the parser. This stratification was conducted to ensure sufficient cases for assessing various performance metrics, particularly precision and recall. All progress notes from all available encounters were included in the sample.

**Table 1:** Description of the stratification variables applied to study sample.

Variable	Description
COVID-19 vaccination	Evidence of COVID-19 vaccination in structured data up to 12 months prior to the start of the study period or during the study period^a^
Influenza vaccination	Evidence of influenza vaccination in structured data during the study period
RSV immunization	Evidence of RSV immunization in structured data during the study period
SARS-CoV-2 test result	Presence of a positive SARS-CoV-2 test result during the study period
Influenza test result	Presence of a positive influenza test result during the study period
RSV test result	Presence of a positive RSV test result during the study period
Age group	Individual's age group (years): 0–9, 10–17, 18–64, 65+
Immunocompromised	Discharge summary contains ICD-10 code(s) for one or more of: hematologic malignancy; solid malignancy; transplant; rheumatologic/inflammatory disorder; or other intrinsic immune condition or immunodeficiency
RSV immunization eligibility^b^	Patients eligible to receive RSV immunization during the study period: age ≥60 years or age ≤9 months or pregnant during the study period

a COVID-19 vaccination looks 12 months prior to the start of the study period as SARS-CoV-2 is a non-seasonal virus.

b RSV immunization eligibility refers to eligibility during the study period.

Stratification by the presence of structured immunization records, testing results, and immunocompromised status was done to assess whether missingness rates — and potential biases — varied across these variables. Section 3.4 discusses a few identified differences in missingness rates. Methods for estimation and adjustment for potential biases are topics for future work.

### 2.3 Algorithm development

We developed the initial algorithm iteratively using a combination of synthetic data generated by ChatGPT and publicly available, de-identified clinical data from the MIMIC-IV database (9). The synthetic data were generated using a prompt requesting artificial medical records including patient medical histories, assessment notes, medications, and social history. Further, we requested that some records contain relevant vaccine histories and that other records should have vaccine histories for other viruses, such as HPV. The prompts and example results are provided in the Supplementary Table S3. This data set was manually annotated. The initial algorithm was developed for influenza only due to the absence of COVID-19 and RSV data from the MIMIC-IV database. We extended this algorithm to identify COVID-19 and RSV immunizations and then applied it to the full sample.

**Figure 1:**
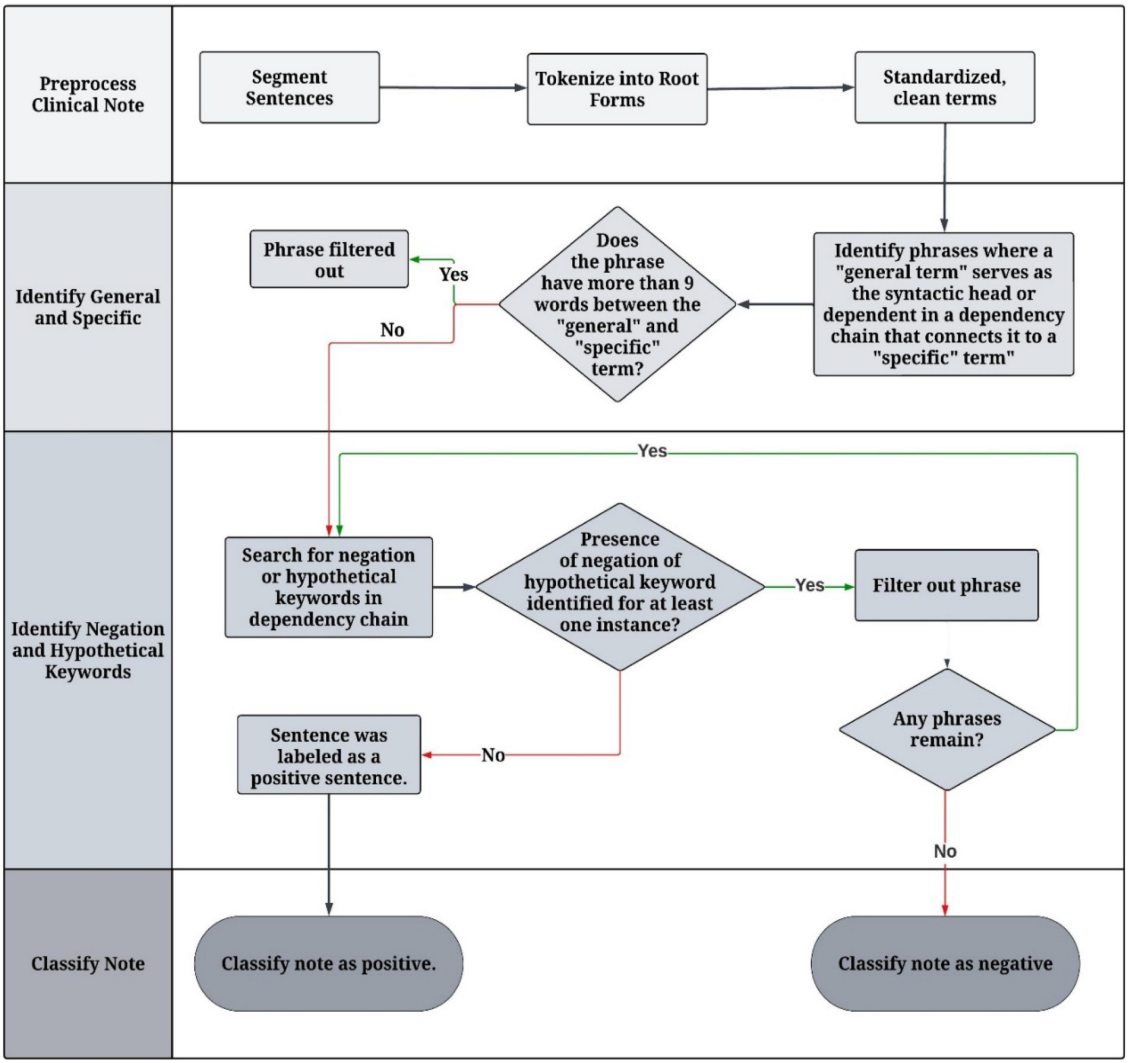
Methodological flow of the study rule-based NLP algorithm to identify immunization evidence in clinical notes.

Our rule-based NLP algorithm used predefined rules and domain expertise to extract immunization history from clinical notes. The algorithm comprised four key components, starting with the preprocessing of clinical notes, which included sentence segmentation, tokenization of sentences into individual words, and lemmatization of each word to its root form (e.g., “vaccinate” to “vaccine”) [Step 1]. Initial evidence of immunization was first identified through a set of general keywords (e.g., “booster”, “shot”, “vaccine”), which were then connected to a “specific term” through grammatical dependencies [Step 2]. The complete list of “general” and “specific” terms is available in Supplementary Table S1. The keywords were then assessed for negation (e.g., “declined”, “refuse”) and hypothetical (e.g., “recommend”, “discuss”) keywords [Step 3]. If none were identified, the sentence was then classified as “positive” [Step 4]. The keyword list for negation and hypotheticals is presented in Supplementary Table S2. Figure 1 describes the NLP process, and Table 2 presents the detailed steps of the algorithm.

**Table 2:** Description of the NLP algorithm procedures for the identification of immunization evidence in clinical notes.

Number	Step	Description
1	Preprocess Clinical Note	Each clinical note was segmented into sentences using the *MedSpaCy library*. After sentence segmentation, text preprocessing was applied to each sentence, which included lowercasing and removing special characters, redundant spaces, and new lines. To prepare for keyword searches in the following steps, the algorithm also tokenized each sentence into individual words and lemmatized each word to its root form (e.g., “vaccinated” to “vaccine”).
2	Perform Named Entity Recognition of Relevant Vaccine Terms	The algorithm applied the *DependencyMatcher* component in *spaCy* to identify instances where a “general” term (e.g., vaccine, shot, booster) that serves as the syntactic head or dependent in a dependency chain that connects it to a “specific” term (e.g., COVID-19, influenza, RSV). To account for lengthy phrases that are difficult to parse correctly, “specific” terms more than nine words apart from the “general” and term were ignored.
3	Identify Negation and Hypothetical Keywords	Once the algorithm identified an instance in step 2, it searched for negation (e.g., decline, refuse, no) or hypothetical (e.g., recommend, discuss, plan) keywords that are associated with the instance in a dependency chain.
4	Classify Note	If no negation or hypothetical keyword was identified for at least one instance, the sentence was labeled as a positive sentence. If at least one sentence within the note was marked as positive, the algorithm classified the note as a positive case.

**Figure 2:**
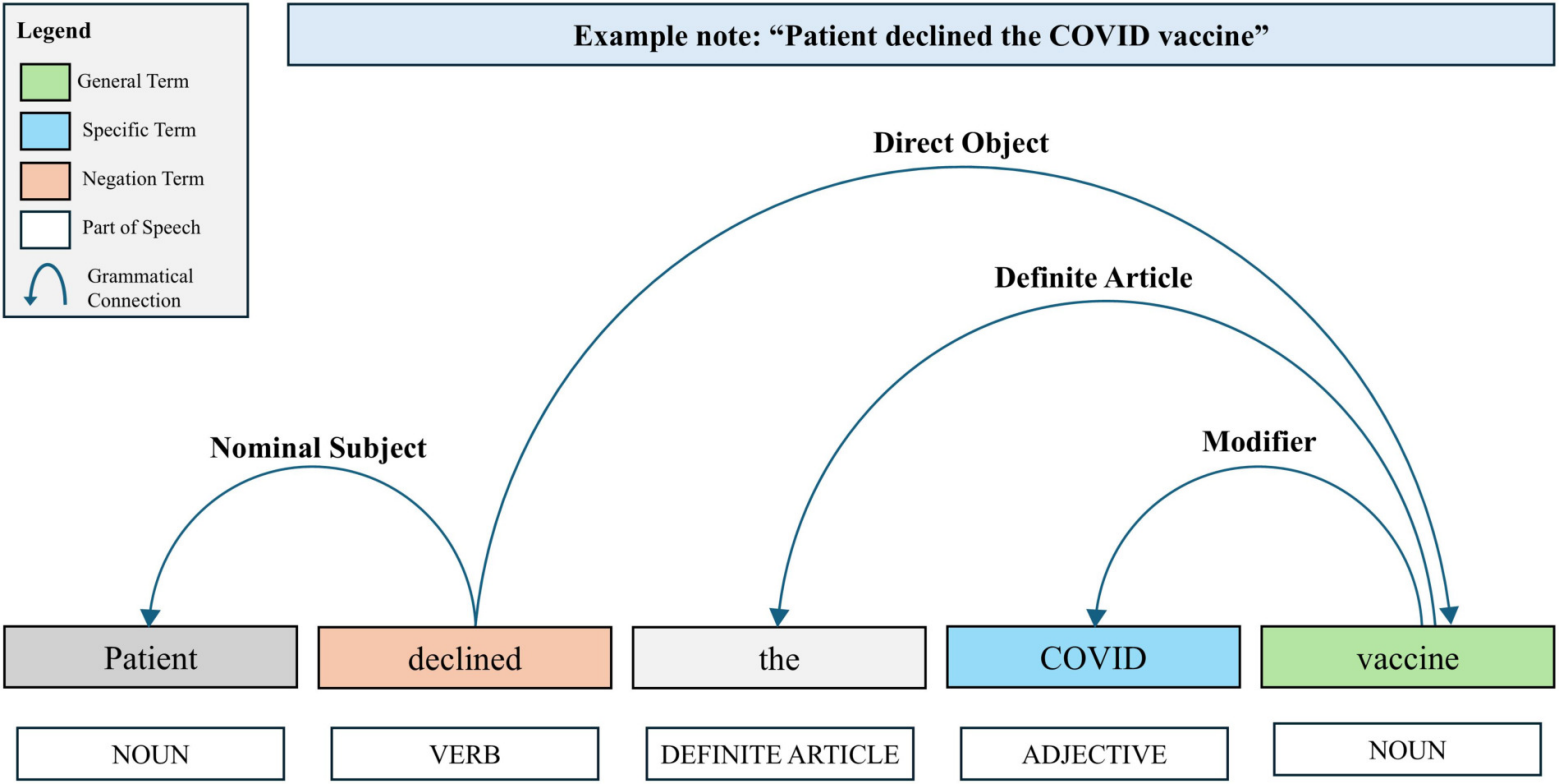
Example of the NLP algorithm dependency parser to capture syntactic dependencies.

Figure 2 illustrates an example of the process with the dependency parser. In the example below, the algorithm identifies the general term “vaccine” and then uses the dependency graph to locate the specific term “COVID” and the negation term “declined”. Thus, this sentence would indicate a negative vaccination. The NLP algorithm was developed using Python version 3.10.12 (33). Text data were processed using Python's *spaCy* library version 3.5.4, an NLP library (34). The *DependencyParser* component of *spaCy* was used to identify syntactic dependencies, and *MedSpaCy* version 1.1.5 was used for sentence segmentation (34).

We developed the algorithm, as described in Figure 1, for COVID-19, influenza, and RSV immunizations and evaluated the results in two ways:

To understand the degree to which the NLP algorithm correctly identified immunization references in the clinical notes, thus assessing the algorithm's overall ability in effectively identifying immunization, we compared the NLP results to immunization history data from a manual review of 400 clinical notes, limited to one note per patient, and calculated performance measures. In this evaluation, the manual review was treated as the gold standard.
To understand the degree to which the NLP algorithm identified immunization administrations absent from the structured data and thus assess the algorithm's overall utility in enhancing immunization detection, we compared the NLP results to immunization history data from the structured sections of the EHR. In this evaluation, the structured data was treated as the gold standard.

We calculated precision and recall for each evaluation. Precision is the probability that an identified immunization is indeed an immunization. High precision suggests the algorithm has a high probability of correctly identifying immunization references when they are present in the notes. Recall is defined as the probability that, if there is an immunization reference, the algorithm identified it. High recall suggests the algorithm has a high probability of capturing immunization references that are present in the notes.

### 2.4 Evaluation based on manual note review

We manually reviewed a stratified random sample of 400 notes (one note for each of 400 patients) selected from the 18,488 patients described in Section 2.2, constructed two-by-two confusion matrices and calculated precision and recall and associated Wilson confidence intervals to identify two types of errors: (1) labeling a patient as having information of interest when the patient's note does not contain that information (false positive), and (2) not identifying existing information of interest that exists in the note (false negative). 109 of the 400 patients were not eligible to receive the RSV vaccine, so were not included in confusion matrices or statistics for RSV. To ensure the reliability of the manual review, two reviewers independently reviewed all progress notes from the 400 patients. To assess reviewer agreement, we calculated Cohen's kappa statistics, which were 0.77, 0.93, and 0.97 for COVID-19, influenza, and RSV, respectively, indicating a relatively strong agreement between reviewers. A third reviewer resolved disagreements between the two reviewers.

We developed a software tool to aid in the manual review of the selected notes. The tool's interface presented the reviewer with the full text of the note, including visual aids and/or annotations based on the NLP algorithm's output. First, the interface would provide the complete clinical note; any immunization administration references identified by the NLP algorithm would be highlighted. For example, if a sentence referenced a COVID-19 vaccination, the phrase would be highlighted. If the NLP algorithm did not identify an immunization reference in the note, the tool highlighted instances of the words “COVID”, “RSV”, “influenza”, “vaccination”, “vaccine”, and “shot” within the note text to help the reviewer identify potential immunization references. To review the note and output, the tool's prompt requested that the reviewer answer questions about each note and leave free-text comments. For example, one prompt asked, “Does the text indicate the patient received COVID vaccination (Yes/No)?” When the reviewer finished with a note, they could continue to the next unreviewed note or return to a previously viewed note. The sample screenshots of the tool's user interface are provided in Supplementary Figures S1a,b.

### 2.5 Comparison with structured EHR data

We constructed two-by-two confusion matrices to assess concordance of the algorithm by respiratory virus and gain insight into the overlap in immunization references between the structured clinical data and information contained in the clinical notes. Concordance was assessed using precision and recall with the structured data as the gold standard. We also calculated the proportion of additional immunizations identified by the NLP algorithm to provide a direct measure of utility. Reported *p*-values were calculated using Pearson chi-square tests.

## 3 Results

### 3.1 Characteristics of the sample

Within the sample (*n* = 20,000 patients), there was nearly equal representation of patients with and without evidence of vaccination in the structured EHR data for COVID-19 and influenza (Table 3). More than half of the sample patients did not have evidence of RSV immunization status (55%) due to the limited number of eligible patients in the sampling frame. Individuals aged 18–64 years comprised approximately half of the sample (42%), while individuals aged 10–17 accounted for the smallest age category in the sample (8%). A majority of the sample was composed of immunocompetent individuals (79%). Of those sampled with testing information, most individuals had a reported negative SARS-CoV-2 molecular test record (75%). Similarly, those with influenza testing information mostly tested negative (85%), and those with RSV testing information also mostly tested negative (95%).

**Table 3:** Characteristics of the sample population (*n* = 20,000) by study stratification variables, the university of Colorado healthcare system, August 1, 2023, to December 21, 2023.

Variable	Level	Count (row %)
COVID-19 vaccination	Present	9,997 (50%)
	Absent	10,003 (50%)
Influenza vaccination	Present	11,127 (56%)
	Absent	8,873 (44%)
RSV immunization	Present	1,767 (9%)
	Absent	7,302 (37%)
	Ineligible	10,931 (55%)
SARS-CoV-2 test result	Positive	4,974 (25%)
	Negative	15,026 (75%)
	Absent	0 (0%)
Influenza test result	Positive	2,360 (12%)
	Negative	13,464 (67%)
	Absent	4,176 (21%)
RSV test result	Positive	315 (2%)
	Negative	7,088 (35%)
	Absent	1,666 (8%)
	Immunization ineligible	10,931 (55%)
Age group	0–9 years old	3,298 (16%)
	10–17 years old	1,529 (8%)
	18–64 years old	8,491 (42%)
	65+ years old	6,682 (33%)
Immunocompromised	Immunocompetent	15,758 (79%)
	Likely immunocompromised	4,242 (21%)
RSV immunization eligibility	Infant (<9 months old)	505 (3%)
	Pregnant	1,882 (9%)
	Older (60+ years old)	6,682 (33%)
	Immunization ineligible	10,931 (55%)

Additionally, absent testing result information was highest in the influenza stratum (21%), followed by RSV (8%). There were no absent testing records for SARS-CoV-2, as the presence of this testing information was prioritized during sampling. The sampling stratification variables and reported frequencies are presented in Table 3, while variable definitions are provided in Table 1.

We attempted to minimize the lack of testing information and RSV immunization ineligibility while targeting an equal distribution of values across other variables; however, due to the limited size of the data frame and the need to balance across several variables, some inequality in representation across strata remained. However, most groups contain over 1,000 patients, and all contain over 300 except for the group with absent SARS-CoV-2 test information (*n* = 0).

### 3.2 Evaluation based on manual note review

**Figure 3:**
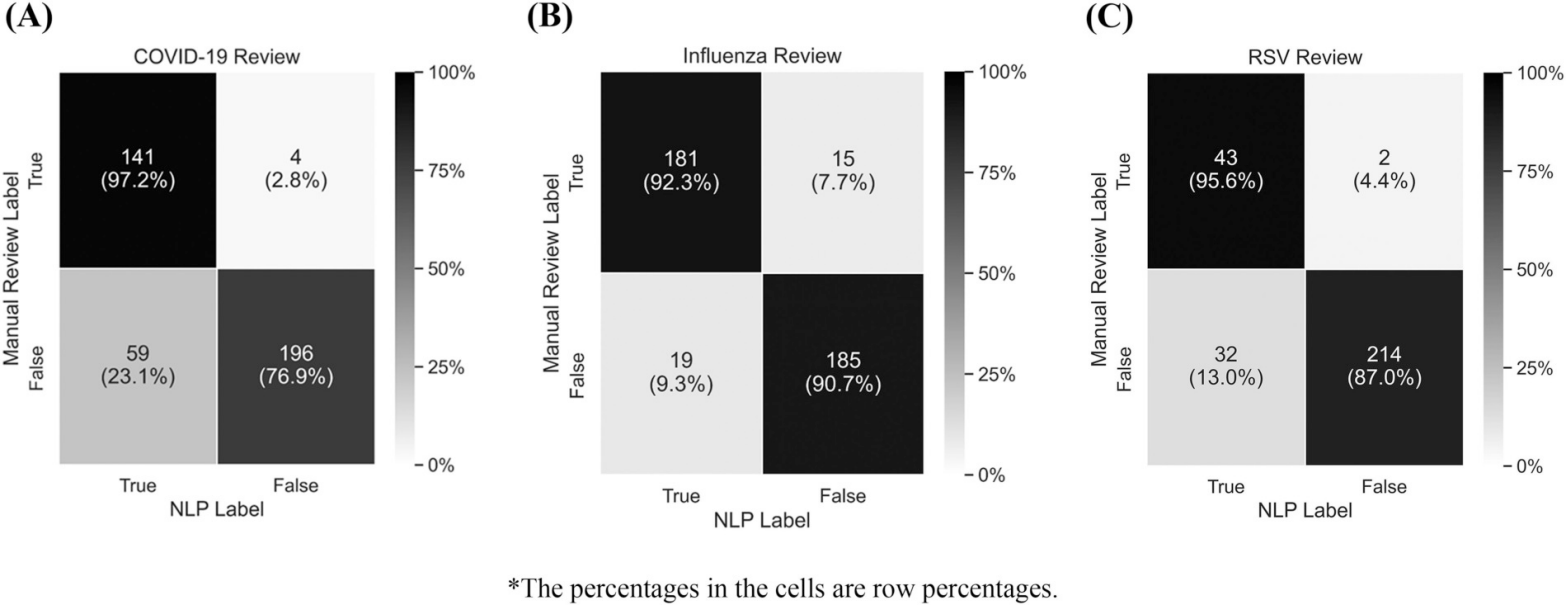
Performance of NLP Algorithm Compared to Manual Clinical Review for the Identification of COVID-19 (A), Influenza (B), and RSV (C) Immunization Evidence*.

Figures 3A–C presents confusion matrices for the evaluation sample. We compared the performance of the algorithm with an independent test dataset using manual clinical review. For COVID-19 immunization, precision was 71% [95% CI: 63%, 77%] and recall was 97% [92%, >99%]; for influenza, precision was 91% [85%, 94%] and recall was 92% [87%, 96%]; and for RSV, precision was 57% [46%, 67%] and recall was 96% [84%, >99%]. Comparatively low precision for RSV may be explained by the prevalence of unrelated respiratory conditions leading to situations where “respiratory” is mentioned in the same context as immunizations but is not in reference to an RSV immunization.

### 3.3 Comparison with structured EHR data

**Figure 4:**
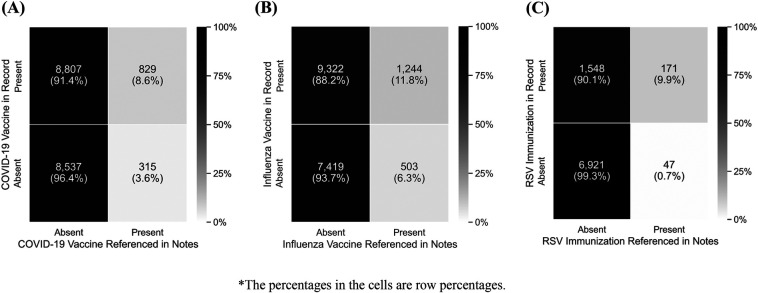
Performance of NLP Algorithm Compared to Structured Electronic Health Record Data for the Identification of COVID-19 (A), Influenza (B), and RSV (C) Immunization Evidence*.

To understand the degree to which the NLP algorithm identified immunization administrations absent from the structured data and thus assess the algorithm's overall utility in enhancing immunization detection, we treated the structured data as the gold standard. Figures 4A–C summarizes these results.

For COVID-19 immunization, precision was 72% [95% CI: 69%, 75%] and recall was 9% [8%, 10%]; for influenza, precision was 71% [69%, 74%] and recall was 12% [11%, 13%]; and for RSV, precision was 78% [72%, 84%] and recall was 10% [8%, 12%]. The NLP algorithm identified patients whose immunization was recorded in the free-text clinical notes but absent from the structured EHR data. The NLP algorithm identified vaccine administrations in 6.3% of cases for influenza, 3.6% of cases for COVID-19, and 0.7% of cases for RSV, among records with no vaccine administration of that type present in structured EHR. Supplementing structured immunization records with these records increased estimated immunization coverage from 52.1% to 53.8% for COVID-19, from 57.1% to 59.8% for influenza, and from 19.8% to 20.3% for RSV.

### 3.4 Immunization identification by strata

Table 4 shows the rate of immunization detection across different patient demographics. For patients without a corresponding immunization in the structured EHR data, COVID-19 and influenza vaccinations were more frequently identified among patients aged 65 years or older compared with other age groups (*p* < 0.001) with detection rates of 5.4% vs. 3.0% for COVID-19 and 1.4% vs. 4.3% for influenza. For influenza, vaccinations were more frequently identified among immunocompromised patients than immunocompetent patients (*p* < 0.001), with detection rates of 5.3% for immunocompromised patients and 3.2% for immunocompetent patients. The NLP algorithm identified RSV immunization references with similar frequencies within each stratum.

**Table 4:** Patient-Level detection of immunization evidence by respiratory virus and patient demographics.

Variable	Evidence of immunization in structured EHR data		No evidence of immunization in structured EHR data	
	Total *N*	Immunization reference found *N* (row %)	Total *N*	Immunization reference found *N* (row %)
SARS-CoV-2				
Overall	9,636	829 (8.6%)	8,852	315 (3.6%)
Immunocompromised status				
Likely immunocompromised	2,542	255 (10.0%)	1,526	81 (5.3%)
Immunocompetent	7,094	574 (8.1%)	7,326	234 (3.2%)
Age				
0–9 years	778	57 (7.3%)	2,046	32 (1.6%)
10–17 years	491	32 (6.5%)	827	18 (2.2%)
18–64 years	4,038	310 (7.7%)	3,801	147 (3.9%)
≥65 years	4,329	430 (9.9%)	2,178	118 (5.4%)
Testing				
Only negative tests in period	8,060	684 (8.5%)	5,935	186 (3.1%)
Positive test in period	1,576	145 (9.2%)	2,917	129 (4.4%)
No test in period	0	0 (N/A)	0	0 (N/A)
Influenza				
Overall	10,566	1,244 (11.8%)	7,922	503 (6.3%)
Immunocompromised status				
Likely immunocompromised	2,533	361 (14.3%)	1,535	166 (10.8%)
Immunocompetent	8,033	883 (11.0%)	6,387	337 (5.3%)
Age				
0–9 years	1,488	14 (0.9%)	1,336	45 (3.4%)
10–17 years	605	66 (10.9%)	713	9 (1.3%)
18–64 years	4,241	459 (10.8%)	3,598	190 (5.3%)
≥65 years	4,232	572 (13.5%)	2,275	259 (11.4%)
Testing				
Only negative tests in period	7,914	949 (12.0%)	4,735	343 (7.2%)
Positive test in period	931	124 (13.3%)	1,162	66 (5.7%)
No test in period	1,721	171 (9.9%)	2,025	94 (4.6%)
RSV				
Overall	1,719	171 (9.9%)	6,968	47 (0.7%)
Immunocompromised status				
Likely immunocompromised	1,138	123 (10.8%)	4,859	30 (0.6%)
Immunocompetent	581	48 (8.3%)	2,109	17 (0.8%)
Age				
0–9 years	42	9 (21.4%)	397	1 (0.3%)
10–17 years	1	0 (0%)	3	0 (0%)
18–64 years	173	18 (10.4%)	1,564	9 (0.6%)
≥65 years	1,503	144 (9.6%)	5,004	37 (0.7%)
Testing				
Only negative tests in period	1,485	150 (10.1%)	5,410	41 (0.8%)
Positive test in period	17	3 (17.6%)	280	0 (0.0%)
No test in period	217	18 (8.3%)	1,278	6 (0.5%)

The results in Table 4 show that the algorithm's effectiveness varied across patient subgroups. The proportion of uncaptured immunizations is higher for certain groups, such as immunocompromised and older patients. Table 5 shows that mentions of vaccine administrations in unstructured clinical notes are more common in outpatient settings than inpatient or urgent care settings.

**Table 5:** Visit-Level detection of immunization evidence by respiratory virus and patient demographics.

Setting^a^	Evidence of immunization in structured EHR data		No evidence of immunization in structured EHR data	
	Total	Immunization reference found	Total	Immunization reference found
	*N*	*N* (row %)	*N*	*N* (row %)
SARS-CoV-2				
Inpatient	89,880	778 (0.9%)	48,182	635 (1.3%)
Outpatient	16,370	1,079 (6.6%)	12,532	445 (3.6%)
Urgent care	10,019	468 (4.7%)	13,608	405 (3.0%)
Influenza				
Inpatient	66,020	1,575 (2.4%)	72,051	2,710 (3.8%)
Outpatient	19,196	1,879 (9.8%)	10,140	522 (5.1%)
Urgent care	11,330	426 (3.8%)	12,213	184 (1.5%)
RSV				
Inpatient	13,562	284 (2.1%)	98,043	1,058 (1.1%)
Outpatient	4,072	170 (4.2%)	13,012	223 (1.7%)
Urgent care	1,308	78 (6.0%)	5,461	37 (0.7%)

a In contrast to other variables which were computed at the patient-level, setting was tabulated at the visit level.

## 4 Discussion

We developed rule-based NLP methods to enhance the identification of COVID-19, influenza, and RSV immunizations from clinical notes, with the goal of supplementing structured EHR data and thereby improving immunization estimates. Based on manual review results, the algorithm identified 97.2% of COVID-19 vaccinations, 92.3% of influenza vaccinations, and 95.6% of RSV immunizations among immunizations recorded in progress notes, with few false positives and slightly more false negatives. Precision varied (70% for COVID-19, 91% for influenza, and 53% for RSV). For COVID-19 and influenza specifically, high precision indicated that when we identified vaccinations in the record, there is a higher probability of being correct. Similarly, high recall suggested that we can identify a high proportion of immunizations in the notes (97% for COVID-19, 92% for influenza, and 96% for RSV). Differences in detection rates across subgroups suggest that our algorithm can identify disparities in how immunization history is documented within structured EHR fields. Conversely, the comparison between immunizations detected by the algorithm and immunizations that are already present in structured data suggests the unstructured text contributed little additional vaccine information beyond what is already captured in the structured data, with potential increases in vaccination coverage limited to 1.7% for COVID-19, 2.7%, for influenza, and 0.5% for RSV. This reinforces our assumptions around the clinical workflow and our understanding of how immunization data are added to unstructured notes. These results are explained by the low recall when treating the structured data as the gold standard, which suggests that there are many more vaccinations in the structured data than are represented in the notes. Taken together, these results suggest that while the algorithm performs well in identifying instances of immunizations in the text, there were relatively few to be found in this application.

As with prior studies, our algorithm performed well and was able to identify relevant clinical terms (i.e., vaccine administrations) when they were present in the data; however, the utility of the algorithm was dependent on the prevalence of those terms in the clinical notes. In contrast to our approach, Deady et al. (10) applied a simpler algorithm limited to the detection of COVID-19, which resulted in a higher number of additional vaccinations being identified (16.8%) despite overall lower performance. We hypothesize that this was due to more robust recording of vaccination in the structured data in the intervening time since that work was done, but this assumption requires more detailed investigation. Similar levels of performance have been achieved in other therapeutic areas. Bucher et al. (15) achieved 95% sensitivity and 86% specificity when using a rules-based approach to identify post-operative surgical site infections. Comparable performance was achieved by Taggart et al. (11), with sensitivity of 85% and positive predictive value of 63%, while Murff et al. (35) in an earlier study achieved slightly lower performance with 77% sensitivity and 63% specificity. Paterson et al. (16) achieved a high level of performance in identifying falls without chart review with precision of 92% and recall of 95%. Rule-based methods have also been shown to be effective in identifying broader mental health and social conditions. Gray et al. (20) identified patients with residential stability issues with 92% precision and 84% recall, while Chandran et al. (19) were successful in disentangling obsessive compulsive disorder from related comorbidities, with 77% precision and 67% recall. These results are consistent with our finding that rule-based NLP methods are effective in identifying clinical terms when they are present in the EHR record.

The performance of our method can be attributed to several key innovations. Our method used grammatical dependencies to understand the relationships between and functions of words in complex sentences, where traditional keyword-based approaches might fail. This allowed us to identify immunization administration in sentences of arbitrary length where the indicator that an immunization was given could be relatively distant from the name of the virus. This advancement proved valuable in accurately interpreting negations and distinguishing between recommended vs. administered immunizations. The effectiveness of our method was further validated by comparing immunizations detected by the algorithm against immunizations recorded in structured data in the EHR. Across the full 20,000 patient sample, the NLP algorithm identified 315 COVID-19 vaccinations, 503 influenza vaccinations, and 47 RSV immunizations that were not captured in the structured data. Due to anticipated differences in immunization uptake across demographic groups, we stratified by immunization status, presence of test result, and immunosuppressed status when calculating immunization detection rates across the full sample. This process enabled us to generate a sample for each immunization that accurately reflected the underlying population, helping us assess the algorithm's performance in a real-world setting. For example, among patients with no evidence of immunization in the structured EHR data, the algorithm detected a higher proportion of influenza vaccinations in immunocompromised patients and older adults, suggesting that it was effective in identifying differences in immunization administration under-capture when they were represented in the clinical notes.

While we have demonstrated our method's performance in identifying immunizations in clinical notes that were not included in structured EHR data, we have not formally evaluated how this performance varies across key analytic variables, including demographics. We hope to perform that evaluation and combine estimates with detection rates across the same variables to estimate the level of completeness in immunization records. One limitation is that most immunized individuals do not have immunization references in clinical notes; however, in addition to supplementing structured EHR data, results on the proportion of patients with immunization references in both notes and structured records can be used to adjust estimates of missingness among patients without immunization references in structured notes. Further research is needed to investigate the impact on VE estimates and explore ways to mitigate biases in these estimates. Failure to capture all vaccine administrations means that some vaccinated individuals would be wrongly included in unvaccinated reference groups during analyses, with the expected effect of underestimating vaccine effectiveness. We also performed preliminary work on identifying the date of immunization; however, due to the appearance of multiple dates in a highly unstructured format, the performance was variable. The accuracy of extracted dates ranged from 20.8% for influenza to 66.7% for RSV. The date of immunization can be a crucial variable in analyses; however, further research is needed to identify administration dates accurately.

In this research, we have developed a generic, keyword-based algorithm that can be applied to multiple clinical data extraction tasks. While our approach has been effective in identifying vaccine administrations when present in clinical text and has addressed some of the limitations of prior methods, more work is needed to extract more complex entities, such as the date of immunization. We have only evaluated it at a single participating institution and applied it solely to identifying immunization characteristics; therefore, its performance remains unclear in more general settings. Future work could apply the algorithm across multiple sites and expand to include other patient characteristics. Other emerging methods that utilize generative AI may capture more complex grammatical structures and semantic similarities necessary to isolate nuanced factors associated with immunization (e.g., date of administration, manufacturer) (36). Therefore, additional work is also needed to quantify and validate the results across different demographics to assess potential bias. If this can be accomplished, our method can be used to estimate missingness in the structured EHR data, which would enhance broader EHR-based analyses.

Due to our specific focus on vaccine administration, we limited our approach to analysis of clinical notes. For more general applications, a potential enhancement would be the incorporation of a broader range of data, such as lab values, imaging studies, and prescription records, which would have the potential to support more complex, multi-modal modelling approaches. Our methods would have more utility if the quality and consistency of clinical documentation was improved, especially for clinical conditions and events that are not well represented by ICD-10-CM or CPT codes, thus suggesting an opportunity to engage with clinicians to improve documentation protocols. A key advantage of our approach is that implementation within the clinical setting is relatively straightforward, requiring only the identification of terms that are typically well-represented in the notes (e.g., disease symptoms), defining of key words through clinical consultation, and the application of the algorithm to the clinical data warehouse. In conclusion, we have shown that rule-based NLP methods are effective in identifying vaccine administrations from clinical text. Due to the ease of implementation they could have utility in general clinical data extraction tasks, especially those that are not reliant on ICD-10-CM diagnosis and procedure codes, which are easily captured in the structured EHR, such as in the identification of adverse events, transfers or referrals, use of treatments and medications, and more nuanced descriptions of disease symptoms.

## Data Availability

The datasets presented in this article are not readily available because data sharing agreements between the CDC and VISION Network partner institutions prohibit making the data publicly available. Requests to access the datasets should be directed to Kevin Wilson, kevinwilson@westat.com.

## Footnotes

^1^Immunization is used throughout this paper to broadly describe vaccination. As non-vaccine products (e.g., monoclonal antibodies) were considered for RSV, when discussing RSV, COVID-19, and influenza, immunization is used; when discussing COVID-19 and influenza, either together or separately, vaccination is used.

^2^See e.g., 45C.F.R. part 46, 21C.F.R. part 56; 42 U.S.C. §241(d); 5 U.S.C. §552a; 44 U.S.C. §3501 et seq.
