# Supplementary material for: Identification and validation of respiratory virus immunization using natural language processing
Source: Front Digit Health. 2026 Feb 2;8:1733630. doi: 10.3389/fdgth.2026.1733630 (PMC12908168; doi:10.3389/fdgth.2026.1733630)
Supplement: Supplementary file 1 [file Table1.docx]

**Table S1. Study Keyword List for the Identification of Immunization Evidence by Virus and Keyword Type by Virus**

| **Virus** | **Type** | **Keyword** |
| --- | --- | --- |
| SARS-CoV-2 | General | adjuvant |
| SARS-CoV-2 | General | astrazeneca |
| SARS-CoV-2 | General | az |
| SARS-CoV-2 | General | biontech |
| SARS-CoV-2 | General | bival |
| SARS-CoV-2 | General | bivalent |
| SARS-CoV-2 | General | booster |
| SARS-CoV-2 | General | bv |
| SARS-CoV-2 | General | comirnaty |
| SARS-CoV-2 | General | immunization |
| SARS-CoV-2 | General | j&j |
| SARS-CoV-2 | General | jab |
| SARS-CoV-2 | General | janssen |
| SARS-CoV-2 | General | jj |
| SARS-CoV-2 | General | jjohnson |
| SARS-CoV-2 | General | johnson |
| SARS-CoV-2 | General | moderna |
| SARS-CoV-2 | General | monoval |
| SARS-CoV-2 | General | monvalent |
| SARS-CoV-2 | General | mv |
| SARS-CoV-2 | General | novavax |
| SARS-CoV-2 | General | pfizer |
| SARS-CoV-2 | General | revaccinate |
| SARS-CoV-2 | General | revaccination |
| SARS-CoV-2 | General | revaccine |
| SARS-CoV-2 | General | series |
| SARS-CoV-2 | General | shoot |
| SARS-CoV-2 | General | shot |
| SARS-CoV-2 | General | spikevax |
| SARS-CoV-2 | General | up to date |
| SARS-CoV-2 | General | up-to-date |
| SARS-CoV-2 | General | utd |
| SARS-CoV-2 | General | vac |
| SARS-CoV-2 | General | vaccinate |
| SARS-CoV-2 | General | vaccination |
| SARS-CoV-2 | General | vaccine |
| SARS-CoV-2 | General | vax |
| SARS-CoV-2 | General | vaxxed |
| SARS-CoV-2 | General | vx |
| SARS-CoV-2 | Specific | biontech |
| SARS-CoV-2 | Specific | comirnaty |
| SARS-CoV-2 | Specific | corona |
| SARS-CoV-2 | Specific | coronavirus |
| SARS-CoV-2 | Specific | covid |
| SARS-CoV-2 | Specific | covid-19 |
| SARS-CoV-2 | Specific | j&j |
| SARS-CoV-2 | Specific | janssen |
| SARS-CoV-2 | Specific | jj |
| SARS-CoV-2 | Specific | jjohnson |
| SARS-CoV-2 | Specific | johnson |
| SARS-CoV-2 | Specific | moderna |
| SARS-CoV-2 | Specific | novavax |
| SARS-CoV-2 | Specific | sars-cov2 |
| SARS-CoV-2 | Specific | spikevax |
| Influenza | General | afluria |
| Influenza | General | astrazeneca |
| Influenza | General | az |
| Influenza | General | fluad |
| Influenza | General | fluarix |
| Influenza | General | flublok |
| Influenza | General | flucelvax |
| Influenza | General | flulaval |
| Influenza | General | flumist |
| Influenza | General | fluzone |
| Influenza | General | glaxosmithkline |
| Influenza | General | gsk |
| Influenza | General | immunization |
| Influenza | General | injectable |
| Influenza | General | jab |
| Influenza | General | medimmune |
| Influenza | General | pasteur |
| Influenza | General | quadrivalent |
| Influenza | General | revaccinate |
| Influenza | General | revaccination |
| Influenza | General | revaccine |
| Influenza | General | sanofi |
| Influenza | General | Seqirus |
| Influenza | General | shoot |
| Influenza | General | shot |
| Influenza | General | up to date |
| Influenza | General | up-to-date |
| Influenza | General | utd |
| Influenza | General | vac |
| Influenza | General | vaccinate |
| Influenza | General | vaccination |
| Influenza | General | vaccine |
| Influenza | General | vax |
| Influenza | General | vaxxed |
| Influenza | General | vx |
| Influenza | Specific | afluria |
| Influenza | Specific | flu |
| Influenza | Specific | fluad |
| Influenza | Specific | fluarix |
| Influenza | Specific | flublok |
| Influenza | Specific | flucelvax |
| Influenza | Specific | flulaval |
| Influenza | Specific | flulaval |
| Influenza | Specific | flumist |
| Influenza | Specific | fluzone |
| Influenza | Specific | influenza |
| Influenza | Specific | lai |
| Influenza | Specific | laiv |
| Influenza | Specific | medimmune |
| Influenza | Specific | seqirus |
| RSV | General | abrysvo |
| RSV | General | adjuvant |
| RSV | General | arexvy |
| RSV | General | beyfortus |
| RSV | General | bival |
| RSV | General | bivalent |
| RSV | General | bv |
| RSV | General | comirnaty |
| RSV | General | glaxosmithkline |
| RSV | General | gsk |
| RSV | General | immunization |
| RSV | General | jab |
| RSV | General | nirsevimab |
| RSV | General | Palivizumab |
| RSV | General | pasteur |
| RSV | General | pfizer |
| RSV | General | revaccinate |
| RSV | General | revaccination |
| RSV | General | revaccine |
| RSV | General | rsvpref3 |
| RSV | General | rsvpref3 |
| RSV | General | sanofi |
| RSV | General | shoot |
| RSV | General | shot |
| RSV | General | up to date |
| RSV | General | up-to-date |
| RSV | General | utd |
| RSV | General | vac |
| RSV | General | vaccinate |
| RSV | General | vaccination |
| RSV | General | vaccine |
| RSV | General | vax |
| RSV | General | vx |
| RSV | Specific | abrysvo |
| RSV | Specific | abrysvo |
| RSV | Specific | arexvy |
| RSV | Specific | beyfortus |
| RSV | Specific | comirnaty |
| RSV | Specific | hrsv |
| RSV | Specific | mab |
| RSV | Specific | monoclonal |
| RSV | Specific | nirsevimab |
| RSV | Specific | orthopneumovirus |
| RSV | Specific | orthopneumoviruses |
| RSV | Specific | palivizumab |
| RSV | Specific | respiratory |
| RSV | Specific | rsv |
| RSV | Specific | rsvpref |
| RSV | Specific | rsvpref3 |
| RSV | Specific | synagix |
| RSV | Specific | syncytial |
