# Supplementary material for: Identification and validation of respiratory virus immunization using natural language processing
Source: Front Digit Health. 2026 Feb 2;8:1733630. doi: 10.3389/fdgth.2026.1733630 (PMC12908168; doi:10.3389/fdgth.2026.1733630)
Supplement: Supplementary file 3 [file Table2.docx]

**Table S2. List of Study Keywords to Account for Negation and Hypotheticals by Relation**

| **Relation** | **Keyword** |
| --- | --- |
| Ancestor of general keyword | afraid |
| Ancestor of general keyword | agreeable |
| Ancestor of general keyword | agreeable |
| Ancestor of general keyword | allergic |
| Ancestor of general keyword | allergy |
| Ancestor of general keyword | anxious |
| Ancestor of general keyword | apprehensive |
| Ancestor of general keyword | avoid |
| Ancestor of general keyword | concern |
| Ancestor of general keyword | consider |
| Ancestor of general keyword | consideration |
| Ancestor of general keyword | decline |
| Ancestor of general keyword | declines |
| Ancestor of general keyword | defer |
| Ancestor of general keyword | deny |
| Ancestor of general keyword | discuss |
| Ancestor of general keyword | due (excluding “due to”) |
| Ancestor of general keyword | encourage |
| Ancestor of general keyword | fearful |
| Ancestor of general keyword | follow up |
| Ancestor of general keyword | follow-up |
| Ancestor of general keyword | incline |
| Ancestor of general keyword | inclined |
| Ancestor of general keyword | miss |
| Ancestor of general keyword | need |
| Ancestor of general keyword | nervous |
| Ancestor of general keyword | no |
| Ancestor of general keyword | no record |
| Ancestor of general keyword | no records |
| Ancestor of general keyword | order |
| Ancestor of general keyword | postpone |
| Ancestor of general keyword | question |
| Ancestor of general keyword | readdress |
| Ancestor of general keyword | recall |
| Ancestor of general keyword | recommend |
| Ancestor of general keyword | recommendation |
| Ancestor of general keyword | reconsider |
| Ancestor of general keyword | refuse |
| Ancestor of general keyword | reject |
| Ancestor of general keyword | reluctant |
| Ancestor of general keyword | remember |
| Ancestor of general keyword | schedule |
| Ancestor of general keyword | should |
| Ancestor of general keyword | status |
| Ancestor of general keyword | suggest |
| Ancestor of general keyword | suggestion |
| Ancestor of general keyword | try |
| Ancestor of general keyword | unclear |
| Ancestor of general keyword | unsure |
| Ancestor of general keyword | wait |
| Ancestor of general keyword | want |
| Ancestor of specific keyword | beside |
| Ancestor of specific keyword | besides |
| Ancestor of specific keyword | excludes |
| Ancestor of specific keyword | expect |
| Ancestor of specific keyword | no |
| Ancestor of specific keyword | not |
| Child of keyword | decline |
| Child of keyword | due (excluding “due to”) |
| Child of keyword | family |
| Child of keyword | follow up |
| Child of keyword | Follow-up |
| Child of keyword | nan |
| Child of keyword | need |
| Child of keyword | no |
| Child of keyword | not |
| Child of keyword | yet |
| Child of root verb | afraid |
| Child of root verb | agreeable |
| Child of root verb | anxious |
| Child of root verb | apprehensive |
| Child of root verb | avoid |
| Child of root verb | before |
| Child of root verb | can |
| Child of root verb | concern |
| Child of root verb | consider |
| Child of root verb | decline |
| Child of root verb | declines |
| Child of root verb | defer |
| Child of root verb | deny |
| Child of root verb | discuss |
| Child of root verb | due (excluding “due to”) |
| Child of root verb | encourage |
| Child of root verb | fearful |
| Child of root verb | follow up |
| Child of root verb | follow-up |
| Child of root verb | if |
| Child of root verb | incline |
| Child of root verb | inclined |
| Child of root verb | may |
| Child of root verb | miss |
| Child of root verb | need |
| Child of root verb | nervous |
| Child of root verb | never |
| Child of root verb | no |
| Child of root verb | not |
| Child of root verb | postpone |
| Child of root verb | question |
| Child of root verb | recommend |
| Child of root verb | refuse |
| Child of root verb | reject |
| Child of root verb | reluctant |
| Child of root verb | should |
| Child of root verb | suggest |
| Child of root verb | to |
| Child of root verb | try |
| Child of root verb | unclear |
| Child of root verb | unsure |
| Child of root verb | wait |
| Child of root verb | want |
| Child of root verb | will |
