# Supplementary material for: Identification and validation of respiratory virus immunization using natural language processing
Source: Front Digit Health. 2026 Feb 2;8:1733630. doi: 10.3389/fdgth.2026.1733630 (PMC12908168; doi:10.3389/fdgth.2026.1733630)
Supplement: Supplementary file 4 [file Datasheet1.docx]

**Figure S1a. User Interface of Developed Study Tool for Manual Review with Example Note Clinical Note**


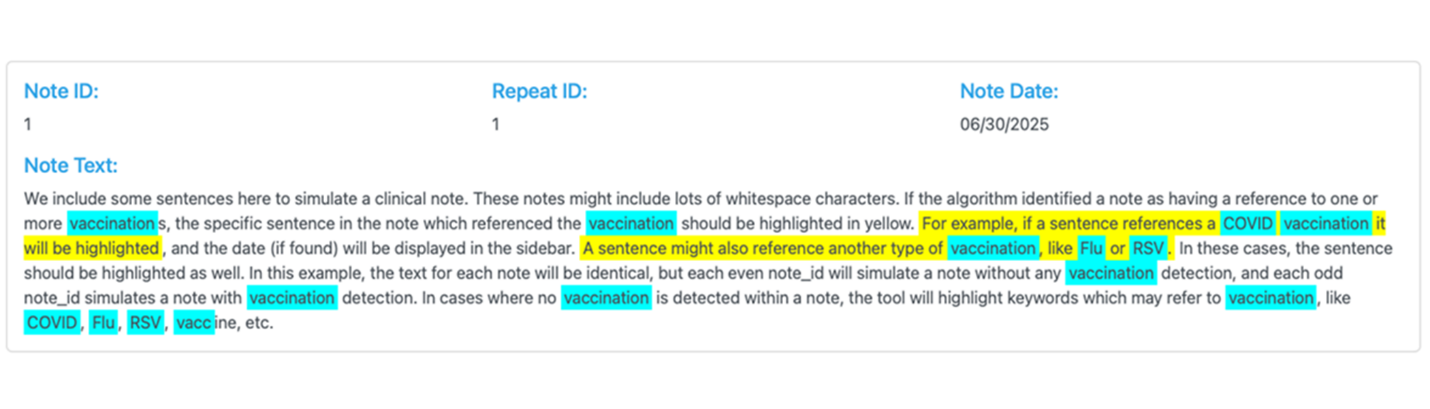


**Figure S1b. Presented Prompts within the Manual Review Tool**

**
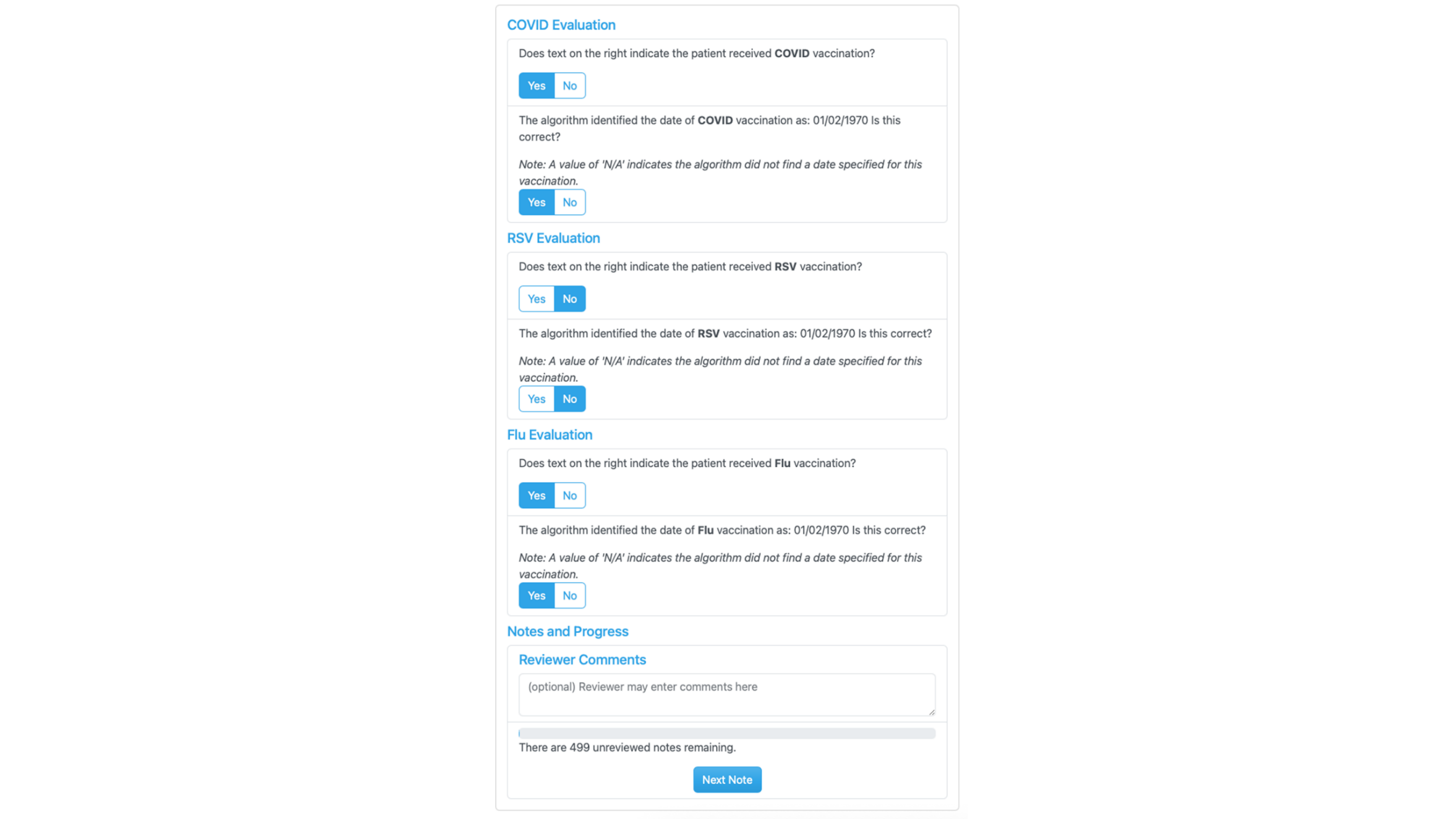
**
